# Variation in human dental pulp stem cell ageing profiles reflect contrasting proliferative and regenerative capabilities

**DOI:** 10.1186/s12860-017-0128-x

**Published:** 2017-02-02

**Authors:** Amr Alraies, Nadia Y. A. Alaidaroos, Rachel J. Waddington, Ryan Moseley, Alastair J. Sloan

**Affiliations:** 1 Mineralised Tissue Group, Oral and Biomedical Sciences, School of Dentistry, College of Biomedical and Life Sciences, Cardiff University, Heath Park, CF14 4XY, Cardiff, UK; 2 Cardiff Institute Tissue Engineering and Repair (CITER), Cardiff University, Cardiff, CF14 4XY UK; 3 Stem Cells, Wound Repair and Regeneration, Oral and Biomedical Sciences, School of Dentistry, College of Biomedical and Life Sciences, Cardiff University, Heath Park, Cardiff, CF14 4XY UK

**Keywords:** Dental pulp, Stem cells, Cumulative population doublings, Telomeres, Cellular senescence, Differentiation, Multi-potency, CD271

## Abstract

**Background:**

Dental pulp stem cells (DPSCs) are increasingly being recognized as a viable cell source for regenerative medicine. Although significant variations in their ex vivo expansion are well-established, DPSC proliferative heterogeneity remains poorly understood, despite such characteristics influencing their regenerative and therapeutic potential. This study assessed clonal human DPSC regenerative potential and the impact of cellular senescence on these responses, to better understand DPSC functional behaviour.

**Results:**

All DPSCs were negative for hTERT. Whilst one DPSC population reached >80 PDs before senescence, other populations only achieved <40 PDs, correlating with DPSCs with high proliferative capacities possessing longer telomeres (18.9 kb) than less proliferative populations (5–13 kb). High proliferative capacity DPSCs exhibited prolonged stem cell marker expression, but lacked CD271. Early-onset senescence, stem cell marker loss and positive CD271 expression in DPSCs with low proliferative capacities were associated with impaired osteogenic and chondrogenic differentiation, favouring adipogenesis. DPSCs with high proliferative capacities only demonstrated impaired differentiation following prolonged expansion (>60 PDs).

**Conclusions:**

This study has identified that proliferative and regenerative heterogeneity is related to contrasting telomere lengths and CD271 expression between DPSC populations. These characteristics may ultimately be used to selectively screen and isolate high proliferative capacity/multi-potent DPSCs for regenerative medicine exploitation.

## Background

Dental pulp stem cells (DPSCs) are increasingly becoming recognized as a viable cell source for the development of effective cell-based therapies. This is due to their accessibility, multi-lineage differentiation capabilities towards osteogenic, chondrogenic, myogenic and neurogenic lineages; and similar regenerative properties to bone marrow-derived cells [1–4]. DPSCs exhibit a fibroblast-like morphology, plastic adherence, express mesenchymal stem cell (MSC) markers (CD73, CD90 and CD105); and thus satisfy the minimal criteria for MSCs [1, 3, 5, 6]. However, similar to bone marrow stem cells, DPSCs isolated from pulpal tissues are recognised to represent a heterogeneous population, with individual isolated clones demonstrating differences in proliferative rates and their abilities to differentiate down particular lineages [1, 5, 7]. Indeed, despite heterogeneous DPSC population expansion being capable of achieving >120 cumulative population doublings (PDs) in vitro, only 20% of purified DPSCs are capable of proliferating beyond >20 PDs. Of these, only two-thirds were able to generate abundant ectopic dentine in vivo, implying that subset DPSC populations differ in their regenerative potential [5, 7]. In vitro, heterogeneous DPSCs can differentiate into osteoblasts, chondrocytes, adipocytes, neurocytes and myocytes, but it has been reported that there are occasions when DPSCs fail to differentiate into adipocytes, chondrocytes and myoblasts; suggested to be a consequence of the potential stem cell niches within dental pulp tissue [1].

Adult stem cells are proposed to exist in a hierarchical arrangement. Pivotal to this model is the mother stem cell, which divides slowly and asymmetrically to yield a replacement mother cell and rapidly dividing transit amplifying (TA) cells [8]. It has been proposed that as TA cells continue to divide, their proliferative capacity is reduced and they become more lineage-restricted. In contrast, newly formed TA cells possess a greater proliferative and multi-differentiation capacity. The presence of TA cells has been suggested to rise within the post-natal dental pulp, which are the first to differentiate into new odontoblast-like cells following cavity-induced injury [9]. Whilst this would indicate a strong role for TA cells in tissue repair and regeneration, the nature, origins or the relationship of DPSC populations with contrasting proliferative capacities to this hierarchical arrangement, have yet to be elucidated.

Another important requirement for the tissue engineering exploitation of stem cells is the considerable in vitro cell expansion required before sufficient cell numbers are obtained for therapeutic use. However, a significant limitation of stem cell therapy is that extensive in vitro cell expansion eventually leads to proliferative decline and cellular senescence, accompanied by altered cellular behaviour and impaired regenerative potential [10]. This feature has been particularly reported for the in vitro expansion of MSCs from human bone marrow, where no more than 4–7 PDs is recommended in preparations for therapeutic use [11]. For most cell types, in vitro expansion and subsequent cellular senescence is a consequence of replicative (telomere-dependent) senescence, characterised by progressive telomere shortening and the loss of telomeric TTAGGG repeats, due to repeated cell divisions [12]. Cellular senescence may also occur through DNA damage by p53, ionizing radiation or oxidative stress (premature or telomere-independent senescence). Either mechanism is associated with the activation of various signalling pathways, including those involving the tumour suppressor genes, p53 and retinoblastoma protein (pRb), via the cyclin-dependant kinase inhibitors, p21^waf1^ and p16^INK4a^, respectively [12]. However, foetal cells, germ lines, stem cells and many tumour cells are established to contain the human telomerase catalytic subunit (hTERT); a reverse transcriptase capable of the complete replication of telomere ends, which plays a major role in counteracting erosion, maintaining telomeric integrity and proliferative lifespan in these cells [13].

Although significant differences in the ex vivo expansion capabilities of individual DPSC populations has been recognized for some time, few studies have addressed the reasons behind these differences and the subsequent impact of such variations on differentiation and regenerative potential. Consequently, this study aimed to examine whether inherent differences in telomere lengths and relative susceptibilities to cellular senescence between individual DPSC populations, contributed to the significant variations in proliferative and differentiation capabilities identified for individual DPSC populations. To achieve this, proliferative lifespans, stem cell marker expression, multi-potent differentiation capabilities and senescence-related marker development, were characterized for individual DPSC populations expanded from individual human teeth derived from young adults with a similar donor age range.

## Methods

### DPSC isolation, culture and population doubling levels

Human third molar teeth were collected from three young adult patients (Patients A-C, all female, age range 18–30 years), undergoing orthodontic extractions at the School of Dentistry, Cardiff University, UK. Teeth were collected with informed patient consent and ethical approval by the South East Wales Research Ethics Committee of the National Research Ethics Service (NRES), UK. The outer surface of the teeth were sterilised with 70% ethanol. Teeth were grooved in the mesial, distal and occlusal regions with a rotary bone saw; and halved. Pulps were removed, minced on a glass slide; and the cells were released from the pulpal tissues by digestion with 4 μg/μl collagenase/dispase (1 ml, Roche, Welwyn Garden City, UK), for 45 min at 37 °C. Pulpal tissues from individual patients were prepared separately. Digests were passed through a 70 μm mesh cell strainer and the eluents collected to obtain single cell suspensions. Cells were centrifuged and further re-suspended in 1 ml culture medium; α-Minimum Essential Medium (αMEM) containing ribonucleosides, deoxyribonucleosides, 4 mM L-glutamine, 100 U/ml penicillin G sodium, 0.1 μg/ml streptomycin sulphate, 0.25 μg/ml amphotericin; and 20% foetal calf serum (all Invitrogen, Paisley, UK), in addition to 100 μM L-ascorbate 2-phosphate (Sigma-Aldrich, Poole, UK).

The present study utilised a fibronectin adhesion assay to preferentially select and isolate immature DPSCs, based on their expression of high β_1_-integrin levels on stem cell surfaces [14]. This fibronectin adhesion isolation method has previously been used successfully for the isolation of tissue-specific, immature stem / progenitor cells from dental pulp [15, 16], oral mucosa [17], cartilage [18]; and bone marrow [16]. Briefly, viable cell counts were calculated and cells seeded onto fibronectin-coated, 6-well plates, at 4000 cells/cm^2^. Plates were subsequently maintained at 37 °C / 5% CO_2_ for 20 min, in accordance with our previously published protocol [15, 16]. Following adhesion, the culture medium and non-adherent cells were removed and replaced with 2 ml αMEM medium. Medium was replenished every 2 days.

Colonies (>32 cells) were allowed to form over 12 days in culture, with individual colonies isolated within cloning rings using 100 μl pre-warmed, accutase (PAA, Velizy-Villacoublay, France); and seeded into 1 well of a 96-well plate. Isolated colonies were subsequently expanded until the numbers were sufficient for seeding into T-75 flasks. Of the DPSC colonies established from Patients A-C, six populations (A1, A2 and A3; B1; C2 and C3) were expanded, with PDs for each DPSC population calculated from cell counts throughout their proliferative lifespans in culture until reaching senescence, as previously described [19]. DPSC senescence was identified when PDs were reduced to <0.5 PDs / week and later confirmed by the increased presence of other senescence-related markers, as described below.

### Telomere length determination

DPSCs at various PDs throughout their proliferative lifespans (A1, B1 and C2 at 8 PDs, A2 at 12 PDs, A3 at 9 PDs and 55 PDs, C3 at 16 PDs), were seeded in T-75 flasks at 5000 cells / cm^2^ and grown to confluence. Genomic DNA was isolated from each DPSC population using QIAmp® DNA Mini Kits (Qiagen, Crawley, UK), according to manufacturer’s instructions; and DNA yields quantified at 260 nm (NanoVue, GE Healthcare, Amersham, UK). Telomere length assessments were performed using the TeloTAGGG Telomere Restriction Fragment Length (TRF) Assay Kit (Roche), according to manufacturer’s instructions. Briefly, each DNA sample (1 μg) was digested with HinF1 / Rsa1 (both 40 U / μl, in Kit), at 37 °C for 2 h. DPSC samples, a digoxigenin (DIG)-labelled, Molecular Weight Marker and a positive DIG-labelled, Control DNA sample (both in Kit), were separated (20 mV, overnight) on 0.8% by agarose gels (Agarose MP, Geneflow, Lichfield, UK); in 1x Tris-acetate-EDTA buffer containing 0.2 μg/ml ethidium bromide (Sigma-Aldrich). Gels were subsequently treated with 0.25 M hydrochloric acid solution (Thermo Fisher Scientific) and rinsed (x2) with water, before treatment (x2) with Denaturation Solution (0.5 M sodium hydroxide, 1.5 M sodium chloride solution), rinsing (x2); and treatment with Neutralisation Solution (0.5 M Tris-HCl buffer, pH 7.5, containing 3 M sodium chloride).

Separated products were transferred onto positively charged nylon membranes (Roche) by Southern blotting; using 20× standard sodium citrate (SSC) buffer (3 M sodium chloride / 0.3 M sodium citrate solution, pH 7.0). Nylon membranes were fixed by UV-crosslinking for 2 × 10 s (Stratalinker, Agilent Technologies, Stockport, UK) and washed with SSC (x2), prior to hybridisation with DIG Easy Hyb (in Kit), for 1 h at 42 °C. This was replaced with Hybridisation Solution mixed with Telomere Probe (2 μl, in Kit); and incubated at 42 °C for 3 h. Membranes were consecutively washed (x2) with 25 ml Stringent Wash Buffers, before incubations in Blocking Solution, Anti-DIG-AP Working Solution, Washing Buffer (x2); and Detection Buffer, respectively (all in Kit). Finally, membranes were incubated for 5 min in Chemiluminescent Substrate Solution (in Kit), before being exposed (30 s) to X-ray film (Hyperfilm^®^, GE Healthcare) and developed.

Average DPSC telomere lengths at each PD were calculated from the Southern blot images, using ImageJ^®^ Software (http://rsb.info.nih.gov/ij/), to quantify the densitometic intensity profile of the entire length of the separated products within each particular lane. In line with TRF Assay Kit instructions, each lane was overlaid along its entire length with a grid consisting of 30 equal squares, where telomere-specific signal was detectable. The background intensity of each lane was calculated by selecting squares within each lane containing no telomere-specific signal and by subtracting these averaged values from the squares containing telomeric signal. For each square containing detectable telomeric signal, the total signal density within each square (OD_i_) and the corresponding molecular weight at the mid-point of each square (L_i_) (based on the DIG-labelled, Molecular Weight Marker bands, kb), were calculated. Average telomere lengths were subsequently calculated, using the following formula, where OD_i_ is the chemiluminescent signal and L_i_ is the length of the telomeres at position i:-

$$ \frac{\Sigma \left({\mathrm{OD}}_{\mathrm{i}}\right)}{\Sigma \left({\mathrm{OD}}_{\mathrm{i}}/\ {\mathrm{L}}_{\mathrm{i}}\right)} $$

### Stem cell and senescence-related marker expression

DPSCs were examined by RT-PCR for the expression of purported stem / progenitor cell markers (CD73, CD90, CD105, CD271) and the absence of the hematopoietic stem cell marker (CD45); at various PDs throughout their proliferative lifespans (1–10 PDs, 11–23 PDs, 24–60 PDs and where applicable, >60 PDs). In addition, DPSCs at early PDs were assessed for the expression of hTERT, whilst expression of the senescence-associated marker genes, p53, p21^waf1^ and p16^INK4a^, were analysed for all DPSCs at PDs throughout their proliferative lifespans. DPSCs were cultured in 6-well plates until ~90% confluence. Total RNA was isolated for DPSCs using an RNeasy Mini Kit (Qiagen), according to the manufacturer’s instructions. RNA was quantified by measurement of 260:280 nm ratio (NanoVue). cDNA was synthesised from 1 μg total RNA, using 5x Moloney murine leukaemia virus (M-MLV) buffer, 0.5 μg Random Primers, 0.625 μl RNasin, 1.25 μl dNTPs (10 mM) and 1 μl M-MLV reverse transcriptase, reconstituted to 25 μl with DNase-free water (all Promega, Southampton, UK, total volume 15 μl). All reactions were performed on a G-storm™ GS1 Thermal Cycler (Genetic Research Instrumentation, Braintree, UK), at 37 °C for 1 h, followed by 95 °C for 5 min.

PCR reactions were established including cDNA (1 μl), 5x Green GoTaq™ Flexi Buffer (5 μl), 25 mM Magnesium Chloride Solution (1 μl), 10 mM PCR Nucleotide Mix (0.5 μl), 5 U / μl GoTaq™ DNA Polymerase (0.25 μl, all Promega), 0.04 μg/μl forward primer and 0.04 μg/μl reverse primer (both 1.25 μl). PCR were performed using the primer sequences and cycling conditions described in Table 1, with β-actin serving as the reference housekeeping gene in all cases. Primer replacement with DNase-free water served as the negative controls. All reaction volumes were made up to 25 μl with DNase-free water. Reactions were run on a G-storm™ GS1 Thermal Cycler, with an initial denaturing step of 95 °C (5 min), followed by 35–40 cycles at 95 °C (1 min), 1 cycle at 55–62 °C (1 min), 1 cycle at 72 °C (1 min); and 1 cycle at 72 °C (5 min). PCR products and a 100 bp DNA ladder (Promega) were separated on 2% agarose gels (125 mV, 45 min), in 1x Tris-acetate-EDTA buffer (above). Gel images were captured under UV light and analysed using a Gel Doc 3000 Scanner and Image Analysis Software (Bio-Rad, Hemel Hempstead, UK).

**Table 1:** Details of the primer sequences and cycling conditions used for RT-PCR

Gene marker	Primer sequence	Annealing temp. (°C)	Cycles	NCBI reference sequence
CD73	F:5′GTCGCGAACTTGCGCCTGGCCGCCAAG-3′ R: 5′-TGCAGCGGCTGGCGTTGACGCACTTGC-3′	65	35	NM_001204813.1
CD90	F:5′- ATGAACCTGGCCATCAGCATCG-3′ R:5′- CACGAGGTGTTCTGAGCCAGCA-3′	55	35	NM_006288.3
CD105	F:5′-GAAACAGTCCATTGTGACCTTCAG-3′ R: 5′-GATGGCAGCTCTGTGGTGTTGACC-3′	65	35	NM_001114753.2
CD271	F:5′- CTGCAAGCAGAACAAGCAAG-3′ R:5′- GGCCTCATGGGTAAAGGAGT-3′	55	35	NM_002507.3
CD45	F:5′-GTGACCCCTTACCTACTCACACCACTG-3′ R:5′-TAAGGTAGGCATCTGAGGTGTTCGCTG-3′	65	35	NM_002838.4
hTERT	F:5′- CGGAAGAGTGTCTGGAGCAA-3′ R:5′- GGATGAAGCGGAGTCTGGA-3′	55	40	NM_198253.2
p53	F:5′- AGACCGGCGCACAGAGGAAG-3′ R:5′- CTTTTTGGACTTCAGGTGGC-3′	55	35	NM_001126118.1
p21^waf1^	F:5′- GGATGTCCGTCAGAACCCAT-3′ R:5′- CCCTCCAGTGGTGTCTCGGTG-3′	60	35	NM_001291549.1
p16^INK4A^	F:5′- CTTCCTGGACACGCTGGT-3′ R:5′- GCATGGTTACTGCCTCTGGT-3′	55	35	NM_001195132.1
OCN	F:5′-GCAGGTGCGAAGCCCAGCGGTGCAGAG-3′ R: 5′-GGGCTGGGAGGTCAGGGCAAGGGCAAG-3′	62	35	NM_199173.4
OPN	F:5′- ATCACCTGTGCCATACCA-3′ R:5′- CATCTTCATCATCCATATCATCCA-3′	55	35	NM_001251830.1
PPARγ	F:5′-GCCATCAGGTTTGGGCGGATGCCACAG-3′ R: 5′-CCTGCACAGCCTCCACGGAGCGAAACT-3′	62	35	NM_138711.3
LPL	F:5′-GCTGGCATTGCAGGAAGTCTGACCAATAA-3′ R:5′-GGCCACGGTGCCATACAGAGAAATCTCAA-3′	55	35	NM_000237.2
β-actin	F:5′-AGGGCAGTGATCTCCTTCTGCATCCT-3′ R:5′- CCACACTGTGCCCATCTACGAGGGGT-3′	65	35	NM_001101.3

### DPSC differentiation capabilities

Based on the distinct proliferative differences identified above between A3 and the other DPSC populations assessed, further studies compared the respective osteogenic chondrogenic and adipogenic differentiation capabilities of A3 (at 30 PDs, 62 PDs, and 75 PDs), versus low proliferative capacity DPSC populations, A1 (17 PDs) and B1 (25 PDs). Differentiation was assessed using commercially available media (OsteoDiff, ChondroDiff and AdipoDiff, Miltenyi Biotec, Bisley, UK), according to manufacturer’s protocols and following supplementation with antibiotics, as above.

For the analysis of osteogenic capabilities, DPSCs were seeded at 4.5x10^3^ cells / cm^2^ in 6- or 12-well plates; and maintained in OsteoDiff medium at 37 °C / 5% CO_2_ for 18 days. Medium was replenished every 3 days. At day 18, cells in 6-well plates were used for the RT-PCR analysis of Runx2, osteocalcin (OCN) and osteopontin (OPN) expression (Table 1), whilst the 12-well plates were washed (x2) with phosphate buffered saline (PBS) and fixed with 4% paraformaldehyde (Santa Cruz, Dallas, USA) for 10 min and washed with PBS (x2). Osteogenic cultures were assessed for mineral nodule deposition detection by staining with 2% alizarin red solution, pH 4.2 (500 μl / well, Sigma-Aldrich), for 3 min. Images representing the extent of alizarin red staining were captured by light microscopy (Eclipse TS100, Nikon UK, Kingston upon Thames, UK), using a digital camera (Canon PC1234, Uxbridge, UK).

Chondrogenic capacity was analysed using single cell suspensions containing 2.5 × 10^5^ DPSCs in 1 ml medium, which were centrifuged to produce cell pellets. Medium was replaced with 1 ml of ChondroDiff medium, prior to re-centrifugation. Pellets were maintained in ChondroDiff medium at 37 °C/5% CO_2_ for 24 days. Medium was replenished every 3 days. At day 24, pellets were washed (x1) with PBS, prior to fixation overnight in 3.7% neutral buffered formalin. Each pellet underwent automated histological processing (Leica ASP300 S, Leica Microsystem, Milton Keynes, UK), through a graded alcohol series and paraffin wax embedding. Sections (5 μm) were cut using a sliding microtome (Leica SM2400, Leica Microsystems), collected onto poly-L-lysine coated slides (SuperFrost, Thermo Fisher Scientific); and dried. Sections were deparaffinised and rehydrate with xylene (10 min), industrial methylated spirits (5 min) and water (5 min). Pellet sections were treated with fast green FCF solution (1:5000, Sigma-Aldrich) for 5 min and washed in 1% acetic acid (Thermo Fisher Scientific). Sections were assessed for proteoglycan content by 0.1% Safranin O staining (Sigma-Aldrich) for 10 min; and washed in 95% ethanol-xylene mixture (1:1). Images representing the extent of Safranin O staining were captured, as above.

For the analysis of adipogenic capabilities, DPSCs were seeded at 7.5 × 10^3^ cells / cm^2^ in 6- or 12-well plates; and maintained in AdipoDiff medium at 37 °C/5% CO_2_ for 21 days. Medium was replenished every 3 days. At day 21, cells in 6-well plates were used for the RT-PCR analysis of adipogenic marker, lipoprotein lipase (LPL) and PPARγ expression (Table 1), whilst the 12-well plates were washed (x2) with PBS and assessed for intracellular lipid-rich vacuole accumulation by Oil Red O staining (500 μl/well, Sigma-Aldrich; 3.5 g/l in isopropanol, mixed 3:2 with double-distilled water), for 10 min. Following excess stain removal with double-distilled water and 60% isopropanol, images representing the extent of Oil Red O staining were captured, as above.

### DPSC morphology and size assessment

Digital images were captured, as above, to assess changes in cellular size and morphology throughout each DPSC’s proliferative lifespan. Random cells (20 cells / image, 3 images / PD) were outlined along their peripheral borders and cellular surface areas calculated, using ImageJ^®^ Software. Surface areas were expressed as an average ± standard error of the mean (SE) for each DPSC population and PD.

### Senescence-associated β-galactosidase staining

DPSCs at defined PDs throughout their proliferative lifespans were seeded into 6-well plates at 5000 cells/cm^2^ and maintained at 37 °C / 5% CO_2_ for 24 h. A Senescence Cell Histochemical Kit (Sigma-Aldrich) was used to detect senescence-associated β-galactosidase activity in these cells, according to manufacturer’s instructions. Culture medium was aspirated and the cells washed (x2) with PBS (in Kit). DPSCs were treated with Fixation Buffer (1.5 ml / well, in Kit) for 7 min and washed (x3) with PBS (1 ml / well). Cells were further treated with Staining Solution (1 ml / well, in Kit) and incubated at 37 °C / 5% CO_2_, overnight. Digital images representing the extent of senescence-associated β-galactosidase staining were captured by light microscopy, as above. Random cells (100 cells in total) were counted and the percentage number of positive β-galactosidase stained cells calculated.

### Statistical analysis

Statistical analysis on the differences in cellular surface area (average ± SE) between DPSC populations throughout their proliferative lifespans, was determined by one-way ANOVA with post-test Tukey multiple comparison analysis, using GraphPad InStat 3 (GraphPad Software, La Jolla, USA). Statistical significance were considered at *p* < 0.05.

## Results

### DPSC isolation, expansion and population doubling levels

**Fig. 1:**
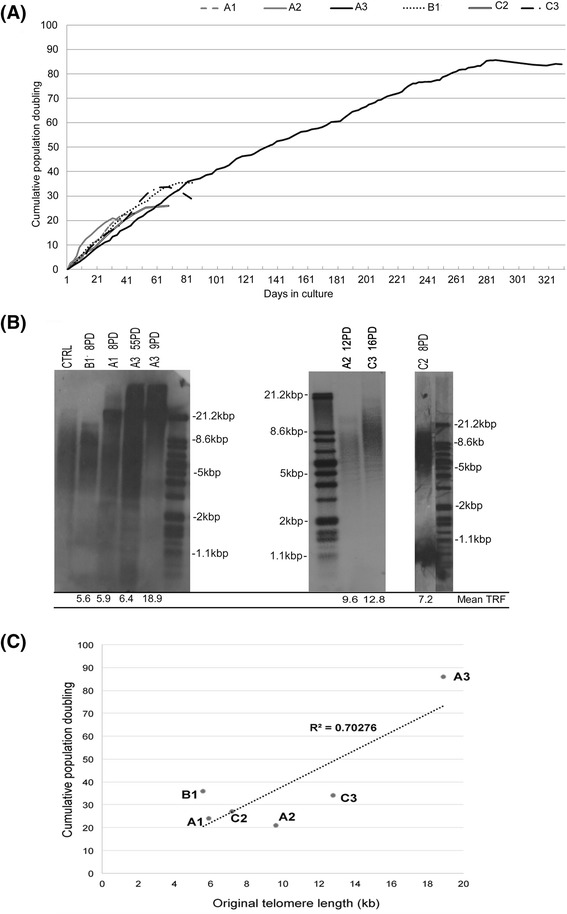
Characterisation of DPSC populations derived from human dental pulp tissues. **a** Cumulative population doublings (PDs) were recorded for six DPSC populations during extended culture until senescence was reached, when PDs reached <0.5 PDs / week. DPSCs A1, A2 and A3 were derived from patient A, while B1, C2 and C3 were derived from patients B and C, respectively. Population A3 demonstrated high proliferative capacity, achieving >80 PDs over 280 days in culture, whilst the other DPSC populations only achieved 20–35 PDs over 35–85 days in culture. **b** Telomere length analysis for the six DPSC populations at cited PDs, by Southern blotting. Average telomere lengths were calculated using ImageJ^®^ Software. Much longer telomeres were identified with high proliferative capacity, A3 (18.9 kb), compared with low proliferative capacity populations, A1 (5.9 kb), A2 (9.6 kb), B1 (5.6 kb), C2 (7.2 kb) and C3 (12.8 kb). A3 (18.9 kb, 9 PDs) only exhibited equivalent telomere lengths to low proliferative capacity DPSCs, at much later PDs towards the end of its proliferative lifespan (6.4 kb, 55 PDs). CTRL lanes represent the separate DIG-labelled, Control DNA sample. Average telomere lengths values obtained for each CTRL lane from left-right, were 10.6 ± 0.73 kb, 11.4 ± 0.71 kb and 11.1 ± 0.60 kb, respectively. Separated DIG-labelled telomere length standards are also included. **c** Correlation between cumulative PDs versus original telomere lengths for all DPSCs analysed

Following the isolation of immature DPSC populations using the fibronectin adherence selection methodology [15, 16], DPSCs were allowed to form colonies of >32 cells over a 12 day period. Of the DPSC colonies established, a selection of six populations (A1, A2 and A3; B1; C2 and C3), were subsequently expanded in vitro through to senescence, confirmed when cumulative PDs fell to <0.5 PDs/week. The cumulative PDs for each DPSC population are presented in Fig. 1a. Marked variations in proliferative capacity were evident between individual DPSC populations, with each demonstrating differences in PDs, irrespective of whether DPSCs were derived from the same or different patients. This was particularly apparent with A3, which demonstrated the highest PD levels (>80 PDs) over 280 days in culture, whilst other DPSC populations from the same patient (A1 and A2) and different patients (B1, C2 and C3) only achieved between 20 and 35 PDs over 35–85 days in culture. In support of the contrasting PDs capabilities between these DPSC populations, telomere length analysis demonstrated much longer average telomere lengths with high proliferative capacity, A3 (18.9 kb), compared with DPSCs with low proliferative capacities at comparable PDs, A1 (5.9 kb), A2 (9.6 kb), B1 (5.6 kb), C2 (7.2 kb) and C3 (12.8 kb) (Fig. 1b and c). The consistency of analysis between separate Southern blots was confirmed by the average telomere lengths calculated for each positive Control DNA sample on each blot (i.e. 10.6 ± 0.73 kb, 11.4 ± 0.71 kb and 11.1 ± 0.60 kb, for the CTRL lanes from left-right in Fig. 1b, respectively).

### Stem cell and senescence-related marker expression

**Fig. 2:**
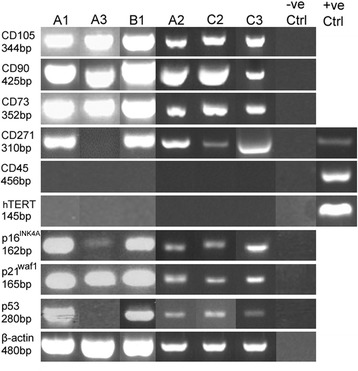
Gene expression analysis of DPSC populations by RT-PCR, at early PDs. All DPSCs showed positive expression for the MSC markers, CD73, CD90 and CD105; and the absence of the hematopoietic marker, CD45. All DPSCs analysed were also negative for hTERT. Only low proliferative capacity DPSCs (A1, A2, B1, C2 and C3) were positive for the expression of CD271 (nerve growth factor receptor p75, LNGFR) and for all 3 senescence-associated marker genes analysed, p53, p21^waf1^ and p16^INK4a^. Replacement of cDNA with H_2_O served as negative controls, with β-actin serving as the reference housekeeping gene

Each DPSC population was further investigated to compare stem/progenitor cell marker expression at early PDs (Fig. 2). All DPSCs demonstrated the expression of the MSC markers (CD73, CD90 and CD105) and the absence of the hematopoietic marker, CD45 [3]. Low proliferative capacity DPSCs (A1, A2, B1, C2 and C3) were also positive for the expression of nerve growth factor receptor p75 (CD271). All DPSCs analysed also demonstrated the expression of the senescence-associated marker genes, p21^waf1^ and p16^INK4a^, at early PDs; although p16^INK4a^ expression appeared to be much less for the high proliferative capacity, A3, than with the low proliferative capacity DPSCs (A1, A2, B1, C2 and C3). Similarly, the other senescence-associated marker investigated, p53, was only expressed in the low proliferative capacity DPSCs (A1, A2, B1, C2 and C3); being undetectable in high proliferative capacity, A3. All DPSCs analysed were negative for hTERT expression.

### DPSC differentiation capabilities

**Fig. 3:**
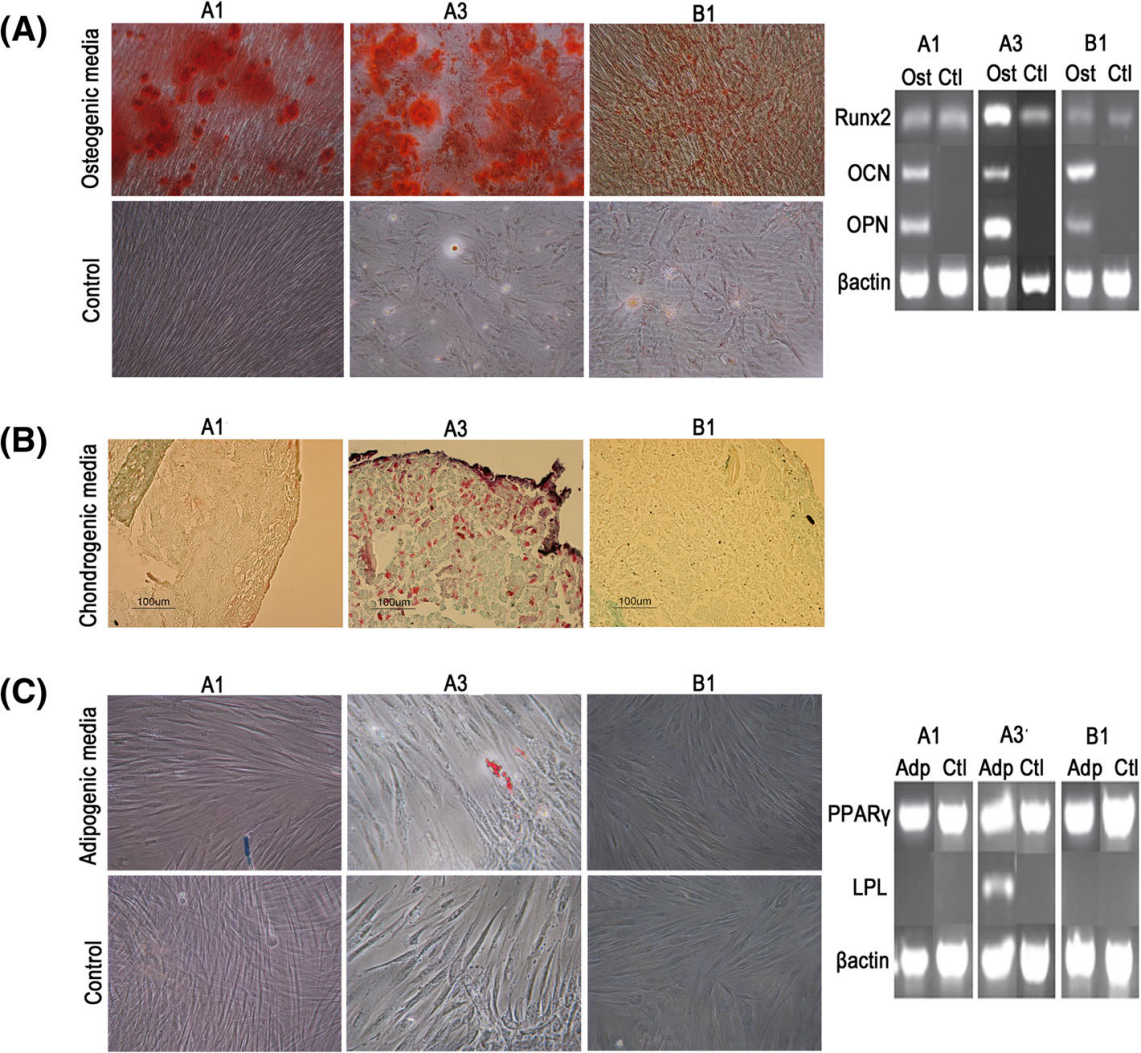
Osteogenic, chondrogenic and adipogenic differentiation of DPSCs. Tri-lineage differentiation analysis was compared between high proliferative capacity, A3 (30 PDs) and low proliferative capacity DPSCs, A1 (11 PDs) and B1 (15 PDs). **a** Osteogenesis was confirmed for each DPSC population by the detection of alizarin red staining for mineralised calcium nodules. No staining was observed in control DPSC cultures in the absence of osteogenic medium. RT-PCR analysis of osteogenic markers (*right panel*) indicated increased osteocalcin (OCN) and osteopontin (OPN) expression for DPSCs cultured in osteogenic media. Cells maintained in both osteogenic and control media expressed Runx2. **b** Chondrogenesis was only particularly evident with high proliferative capacity, A3, which exhibited positive Safranin O staining for high proteoglycan content. No Safranin O staining was observed with the low proliferative capacity DPSCs, A1 and B1. **c** Adipogenesis was only particularly evident with high proliferative capacity, A3, which exhibited positive Oil Red O staining for intracellular lipid-rich vacuole accumulation. No Oil Red O staining was observed with the low proliferative capacity DPSCs, A1 and B1; or in control cultures in the absence of adipogenic medium. RT-PCR analysis of adipogenic markers (right panel) indicated increased expression of the early adipogenic marker, PPARγ, in all DPSCs maintained in both adipogenic and control cultures; whilst the late adipogenic marker, lipoprotein lipase (LPL), was only expressed in high proliferative capacity, A3, in adipogenic media. Scale bars = 100 μm. β-actin served as the reference housekeeping gene

Based on the contrasting PDs identified between A3 and the other DPSCs assessed, further studies evaluated whether these populations also possessed contrasting differentiation capabilities. Tri-lineage (osteogenic, chondrogenic and adipogenic) differentiation was compared between high proliferative capacity, A3 (30 PDs), versus two low proliferative capacity DPSCs (A1 at 11 PDs, B1 at 15 PDs). Only high proliferative capacity, A3, demonstrated multi-potency. Osteogenic differentiation was demonstrated by positive staining with alizarin red and the up-regulation of OCN and OPN after 18 days in osteogenic medium (Fig. 3a). Chondrogenic differentiation was evident by positive Safranin O staining for high proteoglycan content, following 24 days in chondrogenic medium (Fig. 3b). However, mRNA extraction from cartilage pellets was difficult and thus, prevented gene expression analysis. A3 was also able to undergo adipogenic differentiation, evident by the presence of Oil Red O-positive, lipid vacuoles and the up-regulation of the late adipogenic marker, LPL, expression after 21 days in adipogenic medium (Fig. 3c). In contrast, low proliferative capacity DPSCs, A1 and B1, were uni-potent for osteogenic differentiation only and to a lesser extent than high proliferative capacity, A3; as confirmed by alizarin red staining and OCN / OPN expression (Fig. 3a). Of note, all three DPSCs expressed the early osteogenic and adipogenic markers, Runx2 and PPARγ, irrespective of differentiation status (Fig. 3a and c).

### Prolonged in vitro expansion effects on DPSCs

**Fig. 4:**
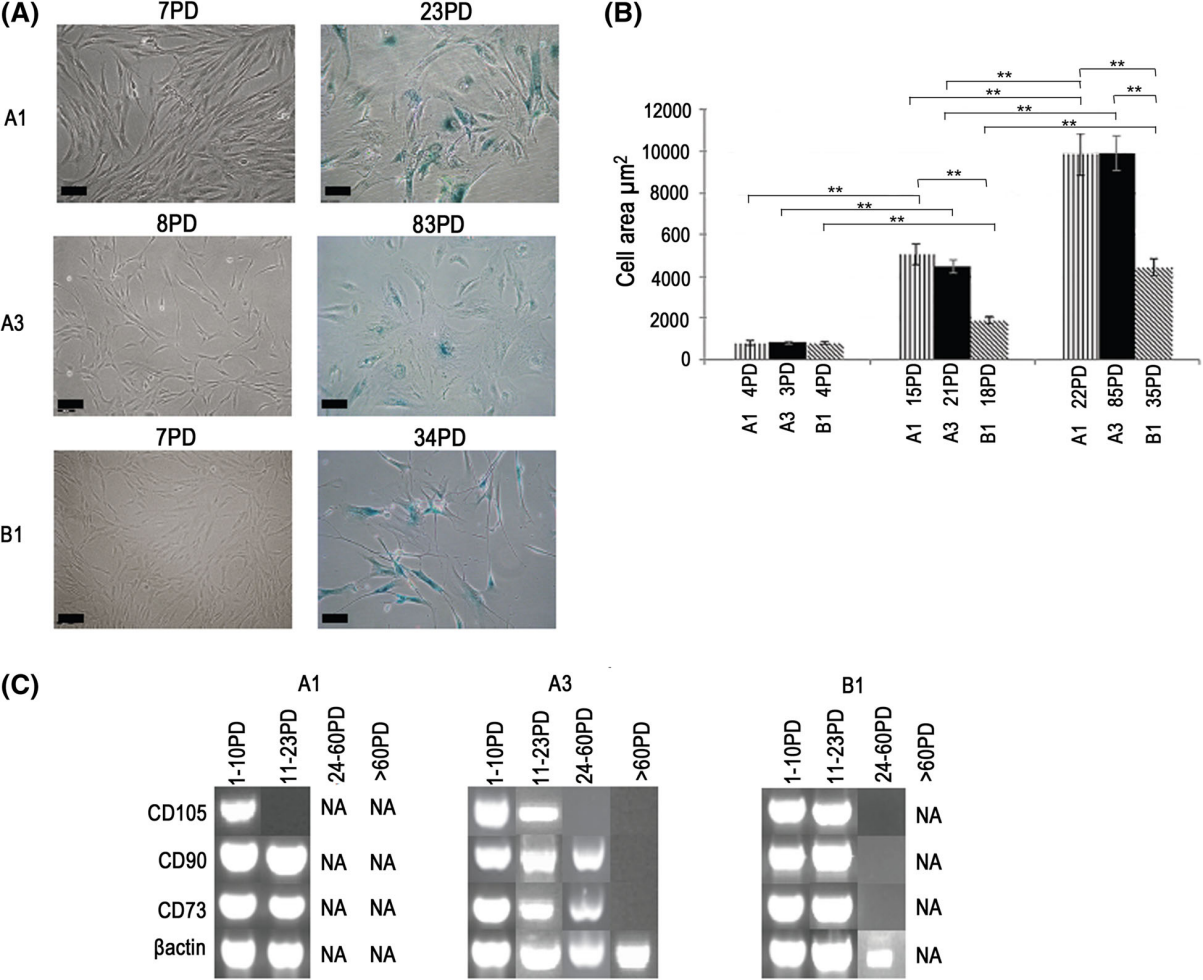
Effects of prolonged in vitro culture expansion on DPSC ageing characteristics. **a** Digital images representing the extent of senescence-associated β-galactosidase staining by DPSCs at early PDs (7–8 PDs) and towards the end of their respective proliferative lifespans (A1 at 23 PDs, A3 at 83 PDs and B1 at 34 PDs). At 7–8 PDs, all DPSCs were morphologically similar, with the characteristic MSC morphology and were negative for β-galactosidase staining. At PDs towards the later stages of their respective proliferative lifespans, higher percentages of cells stained positive for β-galactosidase, which were larger, more stellate-like and with prominent stress fibres. Scale bars = 100 μm. **b** Analysis of cellular surface area throughout the proliferative lifespan of A1, A3 and B1, using ImageJ^®^ Software (average ± SE, **p < 0.001). Significant increases in each DPSC population’s surface area were identified with proliferative lifespan. Only when high proliferative capacity, A3, reached senescence (85 PDs) were surface areas non-significantly different to senescent A1 populations at 22 PDs (*p* > 0.05). Senescent B1 surface areas at 35 PDs did not reach the cell area sizes of senescent A1 or A3 at 22 PDs and 85 PDs, respectively. **c** Gene expression of the MSC markers, CD73, CD90 and CD105, was gradually lost during in vitro expansion. CD105 was lost initially, based on expression levels in A1 (11 PDs) and A3 (24 PDs), whilst CD90 and CD73 expression was subsequently lost towards the end of each population’s respective proliferative lifespans. Whilst the premature loss of CD105 was not as apparent with B1, all three markers were lost towards the end of its proliferative lifespan (24 PDs). NA indicates that no cells were available for analysis at these PDs. β-actin served as the reference housekeeping gene

A decline in cellular proliferative capacity is well-established to correlate with the onset of cellular senescence; and the increased presence of characteristics associated with cellular ageing. At early (7–8 PDs), all DPSCs were morphologically similar, with the characteristic long, spindle, bipolar shape; and were negative for senescence-associated β-galactosidase staining (Fig. 4a). Conversely, at PDs towards the later stages of their respective proliferative lifespans (examples shown for A1 at 23 PDs, A3 at 83 PDs and B1 at 34 PDs), higher percentages of cells stained positive for β-galactosidase and were larger and more stellate-like in appearance, with prominent stress fibres (Fig. 4a). This was confirmed by cell surface area measurements (Fig. 4b), which demonstrated significant increases in the surface areas of DPSCs, A1 and A3, with proliferative lifespan (for A1, approximately 6-fold between 4 PDs and 15 PDs, 12-fold between 4 PDs and 22 PDs; for A3, approximately 6-fold between 3 PDs and 21 PDs; and 12-fold between 3 PDs and 85 PDs; all *p* < 0.001). However, only when high proliferative capacity, A3, had reached senescence at 85 PDs did it exhibit similar non-significant, cell surface area values to senescent low proliferative capacity, A1 populations, at 22 PDs (*p* > 0.05). B1 also demonstrated similar trends of increasing cell surface area with proliferative lifespan, with significant increases in the surface areas with proliferative lifespan (approximately 2.5-fold between 4 PDs and 18 PDs; and 5-fold between 4 PDs and 35 PDs (both *p* < 0.001). However, senescent B1 surface areas at 35 PDs did not reach the cell area sizes of senescent A1 or A3 at 22 PDs and 85 PDs, respectively.

Although the stem/progenitor cell markers, CD73, CD90 and CD105, were previously shown to be expressed by DPSCs, A1, A3 and B1, at early PDs, the expression of these markers was gradually lost during in vitro expansion (Fig. 4c). CD105 appeared to be lost initially, based on expression levels in A1 (11 PDs) and A3 (24 PDs). CD90 and CD73 expression was subsequently lost from these DPSC populations towards the end of their respective proliferative lifespans. Whilst the premature loss of CD105 was not apparent with B1, all three markers were lost towards the end of its proliferative lifespan.

### Prolonged in vitro expansion effects on DPSC differentiation

**Fig. 5:**
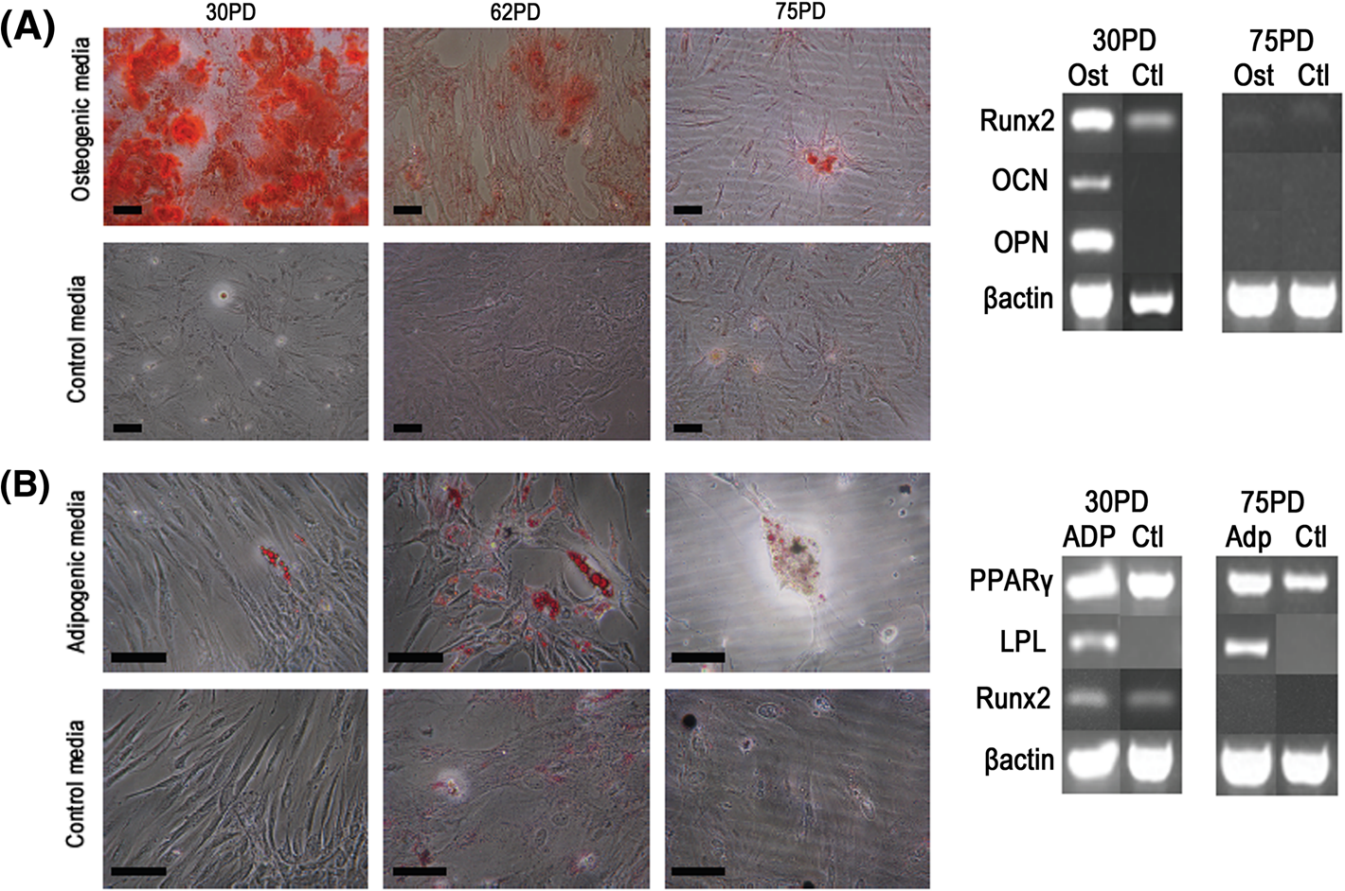
Effects of prolonged in vitro culture expansion on DPSC differentiation capabilities. **a** Osteogenic differentiation potential of high proliferative capacity, A3, was greatly reduced following culture expansion from 30 PDs, to 62 PDs and 75 PDs; apparent by a reduction in alizarin red staining and the loss of osteogenic marker Runx2, osteocalcin (OCN) and osteopontin (OPN) expression (*right panel*). **b** Loss of A3 osteogenic differentiation potential was concomitant with the increased presence of enlarged intracellular lipid vesicles (Oil Red O staining) and the increased expression of early and late adipogenic markers, PPARγ and lipoprotein lipase (LPL, right panel). No staining was observed in control cultures in the absence of osteogenic or adipogenic media. Scale bars = 100 μm

In light of the superior proliferative capacity and multi-potent differentiation properties of the A3 population at early PDs, we also determined whether these preferential differentiation capabilities were diminished with prolonged in vitro expansion, in line with those of low proliferative capacity DPSCs, A1 and B1. A3 telomere lengths decreased from 18.9 kb (9 PD) to 6.4 kb (55 PD) (Fig. 1b). In line with such decreases in telomere length, the osteogenic potential of A3 was greatly reduced following culture expansion from 30 PDs to 62 PDs and 75 PDs, apparent by a reduction in alizarin red staining and the loss of osteogenic marker, Runx2, OCN and OPN, expression (Fig. 5a). However, subsequent analyses indicated that the clear loss of A3 osteogenic differentiation potential at 75 PDs, was concomitant with the increased presence of enlarged intracellular lipid vesicles (Oil Red O staining) and the increased expression of early and late adipogenic markers, PPARγ and LPL (Fig. 5b).

## Discussion

Similar to other tissue sources such as bone marrow, MSC populations in dental pulp are recognised to be heterogeneous populations, with isolated DPSCs demonstrating differences in proliferative rates and ability to differentiate along various lineages. The present study was successful in isolating DPSCs from adult human dental pulp tissues, in order to characterize the relative expansion potentials of individual DPSC populations through to cellular senescence; and the impact of these proliferative lifespan differences on stem/progenitor cell characteristics and multi-potent differentiation potential. Although many animal and human studies have established the effects of increasing donor chronological age on the impairment of stem/progenitor cell regenerative capabilities [20–24], to our knowledge, this is the first study to report inherent differences in the telomere lengths of individual DPSC populations from within a young donor age group (18–30 years); and their correlations to the distinct differentiation capabilities of each population. Indeed, despite DPSCs from young adults with similar donor age ranges having previously been reported to exhibit no significant differences in proliferative or differentiation potential [23, 25], this study identified differences between high (A3) and low (A1 and A2) proliferative capacity DPSC populations, even from the same pulpal tissue sample.

DPSC population, A3, possessed an extensive proliferative capacity (>80 PDs), which was 2-4× the proliferative lifespans of other DPSC populations expanded (20–35 PDs). In support of these findings, A3 was further shown to be morphologically smaller, possessed fewer senescence-associated β-galactosidase positive cells and lacked the expression of p53 and p16^INK4a^, at PDs where low proliferative capacity DPSCs (A1, A2, B1, C2 and C3) demonstrated increased detection of these senescence-associated markers. Such conclusions are consistent with only 20% of purified DPSCs being able to proliferate beyond 20 PDs, whilst it can be surmised that high proliferative capacity DPSCs, such as A3, are responsible for the extensive expansion potential of heterogeneous DPSC populations (>120 PDs) in vitro [5, 7], as DPSCs with less proliferative potential are selectively lost from the mixed population during extended sub-culture [15, 16]. A consequence of A3 possessing a superior proliferative lifespan was the retention of stem cell characteristics, evident by the maintenance of marker expression (CD73, CD90 and CD105) for longer periods in culture (≥23 PDs), compared to the other DPSCs analysed. A3 was also the only population capable of multi-potent osteogenic, chondrogenic and adipogenic differentiation, at least up to 30 PDs in culture. In contrast, other DPSCs appeared lineage-restricted to osteogenic differentiation only. Therefore, there appears to be a clear association between DPSC PDs and the relative differentiation abilities of these populations. However, as DPSCs derived from adult teeth are generally regarded to possess lower proliferation rates and longer population doubling times than those isolated from human exfoliated deciduous teeth (SHEDs) [26–29], it is conceivable that SHEDs are still likely to have equivalent or even greater proliferative potential than adult DPSCs.

The principle reason identified to be behind these contrasting proliferative responses were the average telomere lengths of the DPSC populations. Although it is possible that these differences reflect the relative number of cell divisions undertaken by each stem cell before isolation, such contrasting telomere lengths may also suggest the existence of sub-populations of DPSCs within the stem cell population, with superior telomere dynamic characteristics. Indeed, at early PDs, A3 (18.9 kb) demonstrated telomere lengths 1.5–3.5× longer than low proliferative DPSCs (~5–13 kb). A3 only exhibited equivalent telomere lengths to those of the low proliferative DPSCs at much later PDs towards the end of its proliferative lifespan (6.4 kb, 55 PDs), corresponding to the increased presence of senescence-associated markers. Actively proliferating stem cells are well-known to possess longer telomeres (10–20 kb), compared to somatic cells (5–15 kb) [30], whilst previous studies have reported DPSC telomere lengths between 9.4 and 12.1 kb [31]. Therefore, the contrasting telomere lengths determined for high and low proliferative capacity DPSCs are generally in line with those previously identified in MSCs from other sources.

Based on the correlation comparison between cumulative PDs versus original telomere lengths for all DPSCs analysed, it appears that this relationship is a little more complex than expected, particularly between the low proliferative capacity DPSCs (A1, A2, B1, C2 and C3). For instance, C3 (12.8 kb) had an initial telomere length 2–3× longer than A1 (5.9 kb) and B1 (5.6 kb), yet exhibited similar proliferative capacities (20–35 PDs). The extrinsic and intrinsic factors which influence the respective telomere lengths and PD capabilities of individual DPSC populations remain to be elucidated, although inherent differences due to inter-patient donor variation are likely to play a significant role. As this initial proof-of-concept study has only compared 6 separate DPSC populations (5 low proliferative and 1 high proliferative) from 3 separate donors, we acknowledge that the present study is somewhat limited, particularly in terms of number of highly proliferative DPSCs with longer telomeres assessed. Consequently, further research is warranted with much larger numbers of high and low proliferative DPSCs from different donors, in order to fully establish the relationship between telomere lengths and how these impact on the overall PD capabilities and multi-potent differentiation capabilities of individual DPSC populations.

Although certain studies have reported hTERT expression in human DPSCs [31, 32], we and others have shown that human DPSCs possess no or negligible hTERT expression [33–35]. Therefore, it appears that hTERT may not be wholly responsible for maintaining telomere lengths in high proliferative capacity DPSCs or for the significant variations in DPSC proliferative capabilities, implying that other intrinsic telomere protective mechanisms exist [36, 37]. Nonetheless, the later onset of senescence in A3 was supported by the delayed expression of senescence-associated genes, most notably p53 and p16^INK4a^, at PDs where these and the other senescence marker gene (p21^waf1^) assessed, were highly expressed in low proliferative capacity DPSCs. MSC replicative senescence is acknowledged to be a multi-step process driven by p53, which promotes growth arrest by inducing p21^waf1^ expression, thereby inhibiting G_1_-S phase progression [12]. MSC telomere erosion can also initiate pRb / p16^INK4a^ checkpoints, triggering permanent senescent states by preventing pRB phosphorylation and suppressing cell proliferation. Alternatively, senescence mediators, such as p53, p16^INK4a^ and pRb, are also associated with premature/telomere-independent senescence [12]. The proliferative, telomere length reductions and senescence-related marker detection with increasing proliferative lifespan presented herein, firmly suggest that the senescence of high proliferative capacity DPSCs, such as A3, is a consequence of replicative senescence; although it is plausible that both G_1_-S phase inhibition and pRb/p16^INK4a^ checkpoint initiation contribute to A3 senescence [12]. In contrast, as p53 and p16^INK4a^ are also implicated in the onset of premature senescence, we can only speculate at present which is the principle mechanism of early senescence and the reasons underlying the pre-existing shortened telomeres in low proliferative capacity DPSCs, following their isolation and short-term in vitro expansion. Nonetheless, as p53 and p16^INK4a^ have both been demonstrated to be the principal mediators of senescence in MSCs [23, 34, 38, 39], as p21^waf1^ also has a role in maintaining stem cell renewal due to its positive effects on cell cycle progression [40]; the contrasting p53 and p16^INK4a^ expression between high (A3) and low (A1, A2, B1, C2 and C3) proliferative capacity DPSCs further confirms the earlier onset of senescence with less proliferative populations at early PDs, compared to A3.

Although telomere length maintenance is recognized to facilitate cell division in stem cell populations [41], a notable observation within this study was the impact that contrasting telomere dynamics between high and low proliferative capacity DPSCs has on lineage differentiation capabilities. Whilst A3 exhibited multi-potency towards osteogenic, chondrogenic and adipogenic lineages at early PDs, low proliferative capacity DPSCs, such as A1 and B1, were only uni-potent for osteogenesis. Only following extensive culture expansion beyond 30 PDs did the osteogenic potential of A3 decline, with a corresponding increase in adipogenic differentiation. Such findings concur with previous reports of reduced multi-potency in DPSCs and MSCs from other sources, with extensive proliferative expansion and senescence [23, 34, 42, 43]. Evidence indicates that osteogenesis and adipogenesis are inversely regulated by transcription factors, Runx2 and PPARγ, with changes to cellular osteogenic/adipogenic potential with age being a consequence of transcription factor dysregulation [41, 43].

Another key consideration relating to the findings, is whether the contrasting regenerative properties between high and low proliferative capacity DPSC populations reflect their isolation from different mesodermal or neuro-ectodermal origins [44, 45], stem cell niches within the dental pulp [2], or are from the same origin, but are at different stages within the proposed hierarchical model for adult stem cells [8]. Although the nature and origins of these DPSCs within dental pulpal tissues have yet to be fully elucidated, the absence of cell surface marker, CD271 (nerve growth factor receptor p75, LNGFR), expression in high proliferative capacity and multi-potent DPSCs, such as A3, may suggest that such populations are not neural crest- or sub-odontoblast layer-derived, as CD271^+^ cells are regarded as being of neural crest origin and have been located within the cell-rich, sub-odontoblast region of dental pulp [44, 46]. In line with the uni-potentiality of low proliferative capacity DPSCs, CD271 has been proposed to inhibit DPSC adipogenic, chondrogenic, myogenic and osteogenic potential [47, 48], although not all studies have demonstrated complete inhibition of multi-potent differentiation in CD271-expressing DPSCs [44, 45]. Nonetheless, positive CD271 expression in low proliferative capacity DPSCs (A1, A2, B1, C2 and C3), may provide further credence to these cells being more lineage restricted than high proliferative capacity DPSCs, such as A3.

The prominence of low proliferative capacity and less potent DPSC populations in the present study may imply that high proliferative capacity/multi-potent DPSCs with longer telomere and lacking CD271 expression represent a minor population within the heterogeneous DPSCs in dental pulp tissues; meaning that highly proliferative/long telomere DPSC populations are more difficult to identify and isolate from pulpal tissues, although donor variability is also likely to contribute to this. However, the identification of telomere length and CD271 expression differences between high and low proliferative capacity and multi-/uni-potent DPSCs does advocate their use as potential phenotypic biomarkers for the identification and selective isolation of superior proliferative capacity DPSC populations from dental pulp tissues for regenerative medicine purposes. Variations in telomere lengths have previously been used as selective markers for the isolation of particular stem/progenitor cell populations from tissues, such as bone marrow and cartilage [49, 50]. Furthermore, CD271 has recently been identified to be highly expressed in STRO-1^+^/c-Kit^+^/CD34^+^ DPSC populations, possessing slow proliferation rates, reduced stemness and early-onset senescence; compared to their STRO-1^+^/c-Kit^+^/CD34^−^ counterparts [45]. However, despite differences in CD271 expression, both DPSC sub-populations exhibited similar osteogenic, myogenic and adipogenic differentiation, although STRO-1^+^/c-Kit^+^/CD34^+^ DPSCs expressing CD271 demonstrated greater neurogenic lineage commitment. High CD90^+^ expressing DPSCs with low CD271 expression have also been demonstrated to possess superior colony-forming efficiencies, prolonged in vitro proliferative/multi-lineage potential; and enhanced in vivo bone formation capabilities, versus CD90^+^ / CD271^+^ DPSCs [48].

## Conclusions

This study has reiterated the significant variability in the proliferative and differentiation capabilities for individual DPSC populations expanded from individual human teeth from donors within a similar age range; and even identified inherent differences between DPSC populations derived from the same patient. The findings presented also strongly support the use of telomere length and CD271 expression as viable markers of high proliferative capacity and multi-potent DPSC populations. Consequently, if such superior DPSC populations are to be fully exploited for regenerative medicine, we must utilise these and other potential markers of DPSC proliferation and senescence, such as Bmi-1 and SSEA-4 [34, 51]. Such criteria will allow the optimization of population selection by selectively screening and isolating better quality DPSCs from whole dental pulp tissues for in vitro expansion and assessment, aiding the translational development of more effective DPSC-based therapies for clinical evaluation and application.

## Abbreviations

DPSCs: Dental pulp stem cells
hTERT: Human telomerase catalytic subunit
LBL: Lipoprotein lipase
M-MLV: Moloney murine leukaemia virus
MSC: Mesenchymal stem cell
NRES: National Research Ethics Service
OCN: Osteocalcin
OPN: Osteopontin
PBS: Phosphate buffered saline
PDs: Population doublings
PPARγ: Peroxisome proliferator-activated receptor γ
pRb: Retinoblastoma protein
RT-PCR: Reverse transcription polymerase chain reaction
SSC: Sodium citrate
TA: Transit amplifying
TRF: Telomere restriction fragment length
αMEM: α-minimum essential medium
